# Breast cancer patients with lobular cancer more commonly have a father than a mother diagnosed with cancer

**DOI:** 10.1186/1471-2407-11-497

**Published:** 2011-11-28

**Authors:** Carolina Ellberg, Håkan Olsson

**Affiliations:** 1 Department of Oncology, Clinical Sciences, Lund. Lund University 221 85, Lund, Sweden; 2 Department of Cancer Epidemiology, Clinical Sciences, Lund. Lund University 221 85, Lund, Sweden

## Abstract

**Abstract:**

**Background**

The association between lobular breast cancer and family history is not clear. The aim of the study was to possibly identifying new hereditary patterns predisposing for cancer in the different histopathologic subtypes of breast cancer, with focus on patients with lobular breast cancer and cancer in their first degree relatives.

**Methods**

In 1676 consecutive breast cancer patients detailed family history of cancer was related to histopathologic subtype of breast cancer.

**Results**

Patients with lobular breast cancer were found to be significantly positively associated with having a father diagnosed with cancer, OR 2.17 (95% confidence interval (CI) 1.37-3.46). The finding persisted after excluding breast cancer in the family. Ductal breast cancer was associated with having a mother diagnosed with cancer. There was a significant association between lobular breast cancer and having a father with prostate cancer, OR 2.4 (CI 1.1-5.3). The occurrence of having a father with prostate cancer for lobular breast cancer patients was higher in the younger patient group, OR 2.9 (CI 1.1-7.8), and was still high but lost statistical significance in the older patient group, OR 1.9 (CI 0.5-7.4). The association between lobular breast cancer and a father remained significant after excluding fathers with prostate cancer, OR 1.94 (CI 1.20-3.14). Other commonly occurring tumor types in the father included sarcoma and leukemia.

**Conclusion:**

We propose that lobular breast cancer is associated with having a father diagnosed with cancer, most commonly prostate carcinoma. Since the association remained after excluding family history of breast cancer, the association seems independent of classical breast cancer heredity. The association with a father diagnosed with cancer also remained after removing prostate cancer, indicating an independence from prostate cancer as well. The reason for this association is genetically unclear, but could involve sex-specific imprinting.

## Background

Risk factors, both genetic and environmental differ between the histopathological subtypes of breast cancer, one example is parity. It has been shown that increased parity reduced the risk for all subtypes with the exception of medullary breast cancer [1]. Another risk factor, ever-use of HRT is associated with an increased risk of lobular breast cancer [2]. In hereditary breast cancer, patients with BRCA1 [3]-associated breast cancer are often hormone receptor negative and is more often of the medullary type, whereas patients with BRCA2 [4]-associated breast cancer have a mixture of receptor negative and positive tumors and histopathologies [5].

The aim of the study was to further investigate the relationship between patients with different histopathological subtypes of breast cancer and cancer in their first degree relatives to possibly identify new hereditary patterns predisposing for cancer. We focused our study on lobular breast cancer since it is the second most common specified breast cancer subtype and not as well studied as ductal breast cancer. Many studies tend to focus more on molecular groups and as a supplement to them we focused our study on the classical histopathologic subtypes as a base for our study. Lobular breast cancer is the second most common histopathological subtype of breast cancer usually comprising between 5-15% of all cases [6-8]. Previous associations between lobular breast cancer and family history of cancer have been found, especially an association to family history of breast cancer in first degree relatives [9-12]. The familial association of lobular breast cancer could mainly be due to patients with BRCA2 mutation or the occurrence of hereditary diffuse gastric cancer [13]. However, there is a need for further studies of familial cancer patterns in lobular breast cancer, comparing different histopathological types or molecular subtypes.

## Methods

The 1676 breast cancer patients included in this study were all patients seen by one physician between 1^st ^of January in 1980 and 1^st ^of January in 2009. Using a standardized questionnaire filled out by the physician, information regarding previous family history of cancer was obtained, as well as reproductive and hormonal factors. The additional information concerning date of diagnosis, histopathological subtype, tumor biologic factors (including estrogen receptor (ER) and progesterone receptor (PR) status) was collected from pathological reports and hospital records. Pathological slides were not reexamined, but the diagnosis determining treatment was used. Recurrence of cancer, death and confirmation of the diagnosis on the relatives was retrieved from the Regional Cancer registry and census registry, and information regarding germline mutations was obtained from the Oncogenetic high-risk registry. Follow up was performed until 1^st ^of January 2009. Informed consent was obtained from all patients. Ethical approvals for this study were obtained by the Ethical review board at Lund University, reference nr: 110/92 and 108/93.

All statistical analyses were performed using SPSS^© ^17.0. Only pure histopathological subtypes were used. Mixed, rare types and those breast cancer cases where the histopathological subtype was only known to be adenocarcinoma were grouped as "Others" and used as a reference group. Known mutation carriers were excluded from all analyses (n = 19). For all analyses, two-sided p-values ≤ 0.05 was considered statistically significant. Odds Ratios (OR) were estimated with 95% confidence intervals (CI). Because of the exploratory nature of this study no adjustment for multiple testing was made, and all results need to be validated in a different material.

Power analysis was made for ductal and lobular breast cancer. Actual sample size was used for each calculation. The significance level was set to 0.05, and the power to 0.80. For father with cancer the smallest detectable OR for ductal breast cancer was 2.016, and lobular breast cancer 3.436. For mother with cancer the smallest detectable OR for ductal breast cancer was 1.774, and lobular breast cancer it was 3.213. Excluding cases with breast cancer, the smallest detectable OR for ductal breast cancer was 2.176, and for lobular breast cancer 4.629.

Each histopathological tumor type was studied separately in a logistic regression model in relation to the occurrence of cancer in fathers, mothers, brothers and sisters (y/n), no family history of breast cancer, horizontal and vertical family history of breast cancer (y/n), ever-use of HRT (y/n), number of children (linear) and age at diagnosis (linear). A second analysis was performed in a similar manner, but excluding cases with a family history of breast cancer.

In a separate regression model histologic subtype of breast cancer was related to having a father diagnosed with cancer (y/n), ER-positivity (y/n), PR-positivity (y/n), horizontal and vertical family history of breast cancer (y/n) and age at diagnosis (linear). An analysis similar to this was performed where cases with a family history of breast cancer was excluded.

A third logistic regression model was used in which lobular breast cancer was related to first degree relatives with cancer (y/n), both including and excluding cases with breast cancer diagnoses in mothers and sisters.

In the last logistic regression model having a father with prostate cancer was related to ductal and lobular breast cancer (y/n) and age at diagnosis (y/n) in three variants; including all in the first, only patients younger the 60 years of age in the second, and patients equal to or older than 60 years of age in the third model. In a fourth model prostate cancer in father was excluded.

Binomial tests were performed by using the number of individuals in each branch of the family and comparing males with cancer to females with cancer.

## Results

The clinical series of 1676 breast cancer patients consisted of 41% invasive ductal carcinoma, 8% invasive of lobular carcinoma, 2% of medullary carcinoma and 1% of mucinous carcinoma. Others included mixed, rare types and those breast cancer cases where the histopathological subtype was only known to be adenocarcinoma constituted 48%. The overall median age at diagnosis was 56 years old, range 23-89. In the material, 931 patients were diagnosed with tumors of one single invasive histological type, 63 with non-invasive, 545 with mixed tumor types and 137 of the patients had tumors that were classified as unspecified adenocarcinoma. At time of diagnosis, 257 (15%) of the breast cancer patients had a father diagnosed with cancer and 357 (21%) had a mother diagnosed with cancer. When removing breast cancer in mothers, 219 (13%) patients had mothers diagnosed with other cancers. Patients with tubular breast cancer had an increased number of sisters with cancer (21%) [14], whereas lobular breast cancer patients had an increased number of fathers diagnosed with cancer. Ever-use of HRT was most common among patients with tubular and lobular breast cancer, and least common among patients with mucinous breast cancer (Table 1).

**Table 1:** Description of the population based patient material

	**All**	**Ductal**	**Lobular**	**Tubular**	**Medullary**	**Mucinous**	**Other**
	
**Age at diagnosis: **median(range)	56 (23-89)	58 (24-89)	60 (30-84)	55 (35-79)	52 (31-77)	59 (36-79)	55 (23-87)
	N (%)	N (%)	N (%)	N (%)	N (%)	N (%)	N (%)
	
**Number of cases:**	1676	695 (41)	141 (8)	43 (3)	27 (2)	25 (1)	745 (44)
**Age at diagnosis:**							
20-39	147 (9)	57 (8)	7 (5)	4 (9)	4 (15)	2 (8)	73 (10)
40-59	848 (51)	336 (48)	62 (44)	23 (53)	13 (48)	10 (40)	404 (54)
60-69	422 (25)	175 (25)	49 (35)	12 (28)	6 (22)	8 (32)	172 (23)
70-89	258 (15)	127 (18)	23 (16)	4 (9)	4 (15)	5 (20)	95 (13)
	
**Cancer in 1° relatives:**							
No family history of cancer	700 (42)	279 (40)	52 (37)	19 (44)	13 (48)	12 (48)	325 (44)
Child	20 (1)	13 (2)	0 (0)	1 (2)	0 (0)	0 (0)	8 (1)
Brother	83 (5)	47 (7)	10 (7)	0 (0)	0 (0)	3 (12)	23 (3)
Sister:							
-all diagnoses	213 (13)	88 (13)	23 (16)	9 (21)	4 (15)	2 (8)	87 (12)
-not including bc	115 (7)	49 (7)	10 (7)	3 (7)	3 (11)	1 (4)	49 (7)
Father:	257 (15)	121 (17)	38 (27)	7 (16)	2 (7)	4 (16)	151 0)
- Prostate	67 (4)	28 (4)	8 (6)	2 (5)	1 (4)	2 (8)	26 (3)
Mother:							
- all diagnoses	357 (21)	178 (26)	34 (24)	5 (12)	3 (11)	5 (20)	152 (20)
- not including bc	219 (13)	116 (17)	19 (13)	4 (9)	3 (11)	1 (4)	92 (12)
Unknown family history	13 (1)	8 (1)	1 (1)	0 (0)	0 (0)	0 (0)	4 (1)
	
**Family history of bc:**							
No family history of bc	1131 (67)	465 (67)	93 (66)	28 (65)	20 (74)	18 (72)	507 (68)
Vertical family history	381 (23)	162 (23)	30 (21)	8 (19)	5 (19)	5 (20)	171 (23)
Horizontal family history	84 (5)	33 (5)	11 (8)	7 (16)	0 (0)	1 (4)	32 (4)
Cousin family history	67 (4)	27 (4)	6 (4)	0 (0)	2 (7)	1 (4)	31 (4)
	
**Ever-use of HRT:**	328 (19)	137 (20)	39 (28)	12 (28)	4 (15)	3 (12)	133 (18)
	
**Hormone receptor status:**							
ER-positive	744 (44)	352 (51)	90 (64)	13 (30)	1 (4)	13 (52)	275 (37)
ER-negative	351 (21)	154 (22)	14 (10)	6 (14)	16 (59)	5 (20)	156 (21)
ER-unknown	581 (35)	189 (27)	37 (26)	24 (56)	10 (37)	7 (28)	314 (42)
PR-positive	611 (36)	294 (42)	70 (50)	10 (23)	2 (7)	9 (36)	226 (30)
PR-negative	446 (27)	196 (28)	32 (23)	9 (21)	15 (56)	9 (36)	185 (25)
PR-unknown	619 (37)	205 (30)	39 (27)	24 (56)	10 (37)	7 (28)	334 (45)

bc = breast cancer

Associations between lobular breast cancer and having a father diagnosed with cancer, OR 1.76 (CI 1.17-2.63), and between ductal breast cancer and having a mother with cancer, OR 1.13 (CI 1.01-1.65), were found. Among tubular breast cancer patients it was less common to have a mother diagnosed with cancer; OR 0.41 (CI 0.16-1.08). Removing cases with breast cancer diagnoses in mothers did not significantly alter the results, and the association between having a father diagnosed with cancer and lobular breast cancer remained, OR 1.77 (CI 1.18-2.65) (Table 2).

**Table 2:** Breast cancer subtype in relation to parental cancer diagnosis status

a)		Father with cancer, n = 297			Mother with cancer, n = 328		
			
	n	OR	95% CI	p-value	OR	95% CI	p-value
Ductal	630	0.95	0.74-1.12	0.71	**1.13**	**1.01-1.65**	**0.04**

Lobular	137	**1.76**	**1.17-2.63**	**0.006**	1.03	0.66-1.60	0.90

Tubular	43	0.84	0.37-1.93	0.69	0.41	0.16-1.08	0.07

Medullary	26	0.39	0.09-1.65	0.20	0.44	0.13-1.55	0.20

Mucinous	25	0.90	0.30-2.65	0.85	0.91	0.32-2.62	0.87

b)		Father with cancer, n = 297			Mother with cancer, excluding breast cancer, n = 245		
			
	n	OR	95% CI	p-value	OR	95% CI	p-value

Ductal	629	0.95	0.73-1.23	0.70	1.30	0.99-1.71	0.06

Lobular	137	**1.77**	**1.18-2.65**	**0.006**	0.84	0.51-1.41	0.52

Tubular	43	0.85	0.37-1.93	0.69	0.52	0.18-1.48	0.22

Medullary	26	0.39	0.09-1.65	0.20	0.78	0.23-2.63	0.69

Mucinous	25	0.92	0.31-2.72	0.88	0.26	0.03-1.91	0.18

Logistic regression models with histopathologic type of breast cancer as dependent variable related to having a father and/or a mother diagnosed with cancer. a, b) were both adjusted for age at diagnosis, breast cancer heredity, ever-use of HRT and number of children. N = 1661. b) was similar to a) but cases with a mother diagnosed with breast cancer were excluded from the analysis. N = 1661. There was no interaction between having a father and a mother diagnosed with cancer; β = 0.681, p-value = 0.428 (data not shown).

Including ER- and PR-status did not alter the results significantly, the association between lobular breast cancer and having a father diagnosed with cancer remained, OR 2.17 (CI 1.37-3.46). Similar to the other analyses removing cases with family history of breast cancer did not alter the results significantly. Neither of the other histopathologic subtypes of breast cancer was associated to a father diagnosed with cancer (Table 3). The association between lobular breast cancer and a father diagnosed with cancer remained after adding all first degree relatives to the analysis. Exclusion of breast cancer in mothers and sisters resulted in no apparent change of the results. There was no case of a lobular breast cancer patient with a child diagnosed with cancer (Table 4).

**Table 3:** Risk factors related to lobular breast cancer

	All			No family history of bc		
		
	OR	95% CI	p-value	OR	95% CI	p-value
Father with cancer	**2.17**	**1.37-3.46**	**0.001**	**2.20**	**1.22-3.94**	**0.008**
ER-status	**3.52**	**1.76-7.03**	**< 0.001**	**3.78**	**1.67-8.59**	**0.001**
PR-status	0.87	0.52-1.46	0.61	0.82	0.45-1.51	0.53
Horizontal inheritance	1.36	0.58-3.20	0.48			
Vertical inheritance	0.88	0.53-1.47	0.63			

Binary logistic regression model with lobular breast cancer as dependent variable, adjusted for age at diagnosis. N = 1036 and 681, respectively. bc = breast cancer.

**Table 4:** Lobular breast cancer in relation to first degree relatives

	All			No breast cancer in 1^st ^degree relatives		
		
	OR	95% CI	p-value	OR	95% CI	p-value
Father	**1.84**	**1.23-2.74**	**0.003**	**1.83**	**1.23-2.74**	**0.003**
Mother	0.98	0.65-1.49	0.93	0.98	0.65-1.49	0.93
Brother	1.05	0.52-2.09	0.90	1.05	0.52-2.09	0.90
Sister	1.20	0.73-1.96	0.47	1.20	0.73-1.96	0.47

Logistic regression model with lobular breast cancer as dependent variable in relation to first degree relatives diagnosed with cancer, adjusted for age at diagnosis. N = 1642.

In our material the five most common diagnoses in fathers with cancer with a daughter with lobular breast cancer were prostate cancer (6%), leukemia (4%), lung cancer (1%), lower gastrointestinal tract cancer (3%), stomach cancer (2%) and sarcoma (2%). The fathers of lobular breast cancer patients were overrepresented in all groups compared to the fathers of ductal breast cancer patients, except lung cancer. No case of male breast cancer was present in these families (Table 5).

**Table 5:** Cancer diagnoses in fathers stratified based on histological subtype of breast cancer

	Ductal bc	Lobular bc
Prostate cancer	4%	6%
Leukemia	2%	4%
Lung cancer	2%	1%
Stomach cancer	1%	2%
Lower GI-tract cancer	1%	3%
Sarcoma	0%	2%
Other cancer diagnoses	7%	9%

Comparisons of cancer diagnoses in father of patients diagnosed with ductal and lobular breast cancer. Information regarding diagnosis was given by the breast cancer patient at time of her breast cancer diagnosis. bc = breast cancer

An increased probability to having a father diagnosed with prostate cancer was found in lobular breast cancer patients, OR 2.4 (CI 1.1-5.3). To determine whether this association was dependent on age a cut off at 60 years old was introduced. In patients diagnosed before age 60 the effect remained, OR 2.9 (CI 1.1-7.8). Analysis on patients diagnosed 60 years of age and older, the statistical significance of the association was lost, OR 1.9 (CI 0.5-7.4). To determine whether the association between lobular breast cancer and the father having cancer was due to prostate cancer alone, fathers prostate cancer was removed from the analysis. The association between lobular breast cancer patients and having a father diagnosed with cancer remained, OR 1.9 (CI 1.2-2.8) (Table 6).

**Table 6:** Cancer diagnoses in the father related to lobular and ductal breast cancer

	Father with prostate cancer, N = 1676			Father with cancer, not prostate cancer, N = 1597		
		
	OR	95% CI	p-value	OR	95% CI	p-value
Ductal cancer y/n	1.0	0.6-2.0	0.90	0.9	0.7-1.2	0.58
Lobular cancer y/n	**2.4**	**1.1-5.3**	**0.003**	**1.8**	**1.2-2.8**	**0.006**
Other cancers y/n	1.0			1.0		

	Father with prostate cancer, age at diagnosis > 60, N = 677			Father with prostate cancer, age at diagnosis < 60, N = 983		
		
	OR	95% CI	p-value	OR	95% CI	p-value

Ductal cancer y/n	1.4	0.5-4.0	0.55	0.9	0.4-2.0	0.74
Lobular cancer y/n	1.9	0.5-7.4	0.34	**2.9**	**1.1-7.8**	**0.003**
Other cancers y/n	1.0			1.0		

Logistic regression models with father diagnosed with cancer as the dependent variable. a) Relating prostate cancer in fathers to histopathologic subtype of breast cancer, b) relating father diagnosed cancer, excluding cases with a father diagnosed with prostate cancer, to histopathologic subtype of breast cancer, c) relating breast cancer patients younger than 60 years of age at diagnosis that have a father diagnosed with prostate cancer to histopathologic subtype of breast cancer, and d) relating breast cancer patients diagnosed at age 60 and above With a father diagnosed with prostate cancer to histopathologic subtype of breast cancer. a, b, c, d) were all adjusted for age at diagnosis.

In the lobular breast cancer patient group, 41 of the 58 of the fathers diagnoses and age at diagnosis where confirmed. The median age at cancer diagnosis for the fathers of the lobular breast cancer patients was 70 years old. The median age of the father at their daughter's birth was 32 years old. It does not necessarily represent age at first child, since the patients diagnosed with lobular breast cancer must not be the oldest child (Table 7).

**Table 7:** Descriptive

		Family history of breast cancer		
			
	All	Yes	No	Prostate
Fathers age at daughter's birth	32	32	33	31
Fathers age at cancer diagnosis	70	70	67	70
Daughter's age at bc diagnosis	60	60	54	50

Description of the age of the father with a cancer diagnosis, or proband (lobular breast cancer patients with a father diagnosed with cancer) at specific events. Confirmation of the cancer diagnosis was possible in 41 of 58 the fathers. bc = breast cancer.

To eliminate the possibility that the paternal side had an increase of both cancer diagnoses in general and/or breast cancer in particular, compared to the maternal side, both the parents of the father and the mother were studied in all lobular breast cancer patients. On the paternal side 5 grandfathers and 13 grandmothers were diagnosed with cancer, and on the maternal side 4 grandfathers and 12 grandmothers were diagnosed with cancer. There were 5 maternal and 4 paternal grandmothers diagnosed with breast cancer. A binomial test was performed on these data and no significant difference was noted (data not shown).

## Discussion

Our data suggest a new familial pattern in patients with lobular breast cancer, demonstrating an association between lobular breast cancer and cancer in the father. Non-BRCA1/2 high risk breast cancer families have previously been associated with prostate cancer reported by Loman *et al. *[15]. The study by Loman *et al. *was not made with pure lobular breast cancer, rather breast cancer in general. Associations between breast cancer and prostate cancer effects have been reported in three other studies as well [16-18], however no subdivision based on histopathologic breast cancer subtype was made. In a study by Rawal *et al. *[19] no association between prostate cancer and breast cancer was found. Neither of previous studies did subdivide their data based on histopathological types which could explain why they did not identify an association similar to ours.

It is clear that the association between a father with cancer and lobular breast cancer is not affected by a mother diagnosed with breast cancer, since there was no change in the results when removing cases with breast cancer diagnoses. Also, no interaction between the father and mother with cancer was found. Since the model was adjusted for ever-use of HRT, which is associated with an increased risk of lobular breast cancer [2], and number of children the likelihood that the finding is dependent on these factors seems unlikely. However, the power of the study was small because of the small sample size. Adding that to the fact that it is an exploratory study, the results need to be validated in an independent cohort.

The median age at diagnosis of the fathers of the lobular breast cancer patient was close to the median age at diagnosis for men in Sweden, which was approximately 75 years old in 2009 [20]. Several of the fathers were born in the middle or late nineteenth century and if they were diagnosed with cancer at an early age they were not registered. These results are therefore biased due to the fact that information regarding cancer diagnosis in those fathers diagnosed at a younger age could not be confirmed since the Swedish tumor registry was not started until 1958.

Our data indicates that different tumor types of tumors in the father are associated with lobular breast cancer in the daughter. The theory that there might be a genetic cause is strengthened by the fact that it was the younger lobular breast cancer patients that had an increased risk of having a father with prostate cancer, compared to the other histopathologic breast cancer subtypes. Genetically caused breast cancer tends to present earlier than sporadic ones [21]. However, the loss of statistical significance in the older patient group could be due to the sample size, which was smaller in the older patient group. It is important to remember that the association between lobular breast cancer and having a father diagnosed with cancer remained significant even when removing cases with a father diagnosed with prostate cancer. Indicating that this phenomenon is not connected to prostate cancer exclusively and that the remaining diagnoses that were more common amongst fathers of lobular breast cancer patients, such as leukemia, sarcoma and lower GI-tract cancer may be involved as well. In some families, sarcoma and breast cancer is associated with the Li-Fraumeni syndrome. However, none of our families fulfill the criteria for either Li-Fraumeni, or Li-Fraumeni like syndrome. That together with the age at onset of our patients strongly indicate that this is not connected with the Li-Fraumeni syndrome, in which the cancer usually presents at a much earlier age [22,23]. Therefore, the task remains to determine if our finding is due to chance or represents a new syndrome.

There was no difference in cancer diagnoses in second and third degree relatives on the paternal or maternal side of the lobular breast cancer cases indicating that the association is not connected to an imbalance in number of cancer diagnoses on the paternal side. It also indicates that it may only be related to the father, or inherited from the father.

There are several possible theories explaining this phenomenon; one could be imprinting. In that case it could mean that the gene or polymorphism was maternally imprinted and therefore only the father's allele would be expressed, and predispose for cancer in men and breast cancer in their daughters. A recent study by Kong *et al. *[24] found a region at 11p15 with a paternal inheritance associated with breast cancer, which further supports the possibility of allelic imprinting or a peculiar inheritance involving males with cancer and females developing breast cancer. Another possibility could be copy number variation which, when placed in a gene beneficial for cancer development could predispose for cancer in men and breast cancer in women. A third possible explanation could be that it is due to common recessive haplotypes. Which in a heterozygote state predisposes for cancer in men but not women, and in a homozygote state predisposes for breast cancer in women. A fourth explanation could be that it is due to uniparental disomy (UPD) where both alleles, in this case, would be inherited from the father. However, the incidence of UPD has been estimated to be 1/80 000 births [25], which makes this hypothesis rather unlikely since we have several cases of patients with a father with cancer and are nowhere near a rate of 1/80 000.

Patients are referred to the Department of Oncology from the Southern Health Care Region in a population based setting. Since Skåne University Hospital, Lund is responsible for radio- and chemotherapy in the southern health care region of Sweden. The present clinical material was slightly younger at diagnosis compared to the median age at diagnosis in the southern health care region of Sweden. This is most likely because some older cases and cases only operated without adjuvant therapy (stage I) may not be referred to the Oncology clinic of Skåne University Hospital, Lund. Overall we receive around 60% of all breast cancer cases for therapy. Therefore our results may not fully be applicable to stage I or older patients. Bias in patient selection, except for the slightly younger population, as well as chance, seems unlikely. The material was collected under a long time period, reducing the possibility that it was a certain branch of the population included. The material was collected for research purposes, and the questionnaire was filled out by the physician in a structured interview, reducing the possibilities for different types of biases. We decided not to correct for multiple testing since this is an exploratory study [26]. The findings in the present study need to be validated in an independent material.

## Conclusion

Our study has identified a possible father-daughter inheritance pattern in lobular breast cancer, independent of previous family history of breast cancer. These findings need to be validated in other materials in order to later elucidate the possible mechanism behind the association.

## Competing interests

The authors declare that they have no competing interests.

## Authors' contributions

CE participated in the design of the study, the assemblage and the statistical analysis of the data and the drafting of the manuscript. HO participated in the design of the data, the collection of the data, and the statistical analysis of the data. All authors read and approved the final manuscript.

## Pre-publication history

The pre-publication history for this paper can be accessed here:

http://www.biomedcentral.com/1471-2407/11/497/prepub
